# Synaptic Organization of Microglomerular Clusters in the Lateral and Medial Bulbs of the Honeybee Brain

**DOI:** 10.3389/fnana.2016.00103

**Published:** 2016-11-01

**Authors:** Theo Mota, Sabine Kreissl, Ana Carrasco Durán, Damien Lefer, Giovanni Galizia, Martin Giurfa

**Affiliations:** ^1^Department of Physiology and Biophysics, Federal University of Minas GeraisBelo Horizonte, Brazil; ^2^Research Center on Animal Cognition, Université de ToulouseToulouse, France; ^3^Research Center on Animal Cognition, Centre National de la Recherche ScientifiqueToulouse, France; ^4^Department of Neurobiology, University of KonstanzKonstanz, Germany

**Keywords:** vision, microglomeruli, anterior optic tubercle, lateral complex, central complex, GABA, serotonin, honeybee

## Abstract

The honeybee *Apis mellifera* is an established model for the study of visual orientation. Yet, research on this topic has focused on behavioral aspects and has neglected the investigation of the underlying neural architectures in the bee brain. In other insects, the anterior optic tubercle (AOTU), the lateral (LX) and the central complex (CX) are important brain regions for visuospatial performances. In the central brain of the honeybee, a prominent group of neurons connecting the AOTU with conspicuous microglomerular synaptic structures in the LX has been recently identified, but their neural organization and ultrastructure have not been investigated. Here we characterized these microglomerular structures by means of immunohistochemical and ultrastructural analyses, in order to evaluate neurotransmission and synaptic organization. Three-dimensional reconstructions of the pre-synaptic and post-synaptic microglomerular regions were performed based on confocal microscopy. Each pre-synaptic region appears as a large cup-shaped profile that embraces numerous post-synaptic profiles of GABAergic tangential neurons connecting the LX to the CX. We also identified serotonergic broad field neurons that probably provide modulatory input from the CX to the synaptic microglomeruli in the LX. Two distinct clusters of microglomerular structures were identified in the lateral bulb (LBU) and medial bulb (MBU) of the LX. Although the ultrastructure of both clusters is very similar, we found differences in the number of microglomeruli and in the volume of the pre-synaptic profiles of each cluster. We discuss the possible role of these microglomerular clusters in the visuospatial behavior of honeybees and propose research avenues for studying their neural plasticity and synaptic function.

## Introduction

The honeybee *Apis mellifera* constitutes a well-established model for the study of visual processing and learning (Avarguès-Weber et al., 2011, 2012). Extensive behavioral studies have shown that honeybees perceive, learn and memorize colors, shapes and patterns when these visual cues are paired with sucrose reward and that they navigate in their environment using visual cues to find their way back to the hive and to the food sources (Menzel and Backhaus, 1991; Srinivasan, 1994, 2011; Giurfa and Menzel, 1997; Dyer, 2012; Zhang et al., 2012; Avarguès-Weber and Giurfa, 2014). Furthermore, landmarks and celestial cues such as azimuthal position of the sun and polarized light pattern of the sky ensure efficient navigation in a complex environment (Wehner and Rossel, 1985; Rossel and Wehner, 1986; Collett et al., 2003).

The study of the neural bases of visual processing in the honeybee has not achieved the same level of progress compared to the behavioral studies performed in this insect. Bee color vision is trichromatic, based on three photoreceptor types (S, M, L), which peak in the ultraviolet (UV), blue and green region of the spectrum (Autrum and von Zwehl, 1962; Menzel and Blakers, 1976). L-photoreceptors project to the first-order visual neuropil, the lamina, which exhibits a columnar organization, while S and M photoreceptors send long projections directly to the second-order neuropil, the medulla (Menzel and Backhaus, 1991; Hempel de Ibarra et al., 2014). Fibers coming from the anterior part of the lamina project to the posterior medulla while posterior fibers from the lamina project to the anterior medulla (Ribi and Scheel, 1981). Thus, the retinotopic organization is retained but reversed in the medulla, which is also organized into a columnar pattern. The third visual neuropil is the lobula, where columnar stratification and retinotopic organization are preserved only in the distal part (Hertel et al., 1987). The inner chiasm forms the connection between the medulla and the lobula, in which the retinotopic organization is again reversed antero-posteriorly. Both medulla and lobula contain distinct spatial- and color-opponent neurons (Kien and Menzel, 1977; Hertel, 1980; Paulk et al., 2008). Extrinsic medulla and lobula neurons form different tracts connecting these neuropils to the mushroom bodies (MBs), a higher-order processing center of the insect brain (Mobbs, 1984). Furthermore, the medulla and lobula are highly connected to the lateral protocerebrum of the bee central brain (Hertel, 1980; DeVoe et al., 1982; Hertel et al., 1987).

In bees, the lateral protocerebrum can be divided in at least five main regions: the superior lateral protocerebrum, the inferior lateral protocerebrum, the posterior protocerebrum, the lateral horn and the anterior optic tubercle (AOTU; Paulk et al., 2009). Whilst the lateral horn is involved in olfactory processing (Roussel et al., 2014), the other protocerebral regions receive visual input from the medulla and/or lobula and participate in visual processing (Paulk et al., 2009).

The neural organization and connectivity of the AOTU has been recently described in the honeybee brain (Mota et al., 2011, 2013; Zeller et al., 2015). This neuropil is compartmentalized in four distinct subunits (Mota et al., 2011): the dorsal and ventral lobes of the major unit (MU-DL and MU-VL, respectively), the lateral unit (LU) and the ventrolateral unit (VLU). These compartments receive substantial input from the medulla and lobula via the anterior optic tract and send output to the lateral complex (LX) via the tubercle-accessory lobe tract (TALT). Axon terminals of the TALT arborize in two different subregions of the LX: the lateral accessory lobe (LAL) and the bulbs (BU; Mota et al., 2011; Zeller et al., 2015). Additionally, two distinct tracts (ventral and medial inter-tubercle tracts: vITT and mITT) interconnect the AOTUs of both brain hemispheres (Mota et al., 2011). Visual information from the dorsal and ventral parts of the bee eye segregate within different AOTU compartments, both at the level of the input, via the anterior optic tract, and of the output to the contralateral AOTU, via intertubercle tracts (Mota et al., 2011). While the VLU treats visual information exclusively from the dorsal medulla, neural circuits of the MU treat in a segregated manner information from the dorsal and ventral parts of the medulla and lobula (Mota et al., 2011). Stimulation of the compound eye with monochromatic lights (UV, blue and green) and distinct blue-green mixtures induced different signal amplitudes, temporal dynamics and spatial activity patterns, providing evidence for a spatiotemporal segregation of chromatic processing in the AOTU, which may serve for navigation purposes (Mota et al., 2013).

Specific neuronal projections from LU and VLU (the so-called the lower unit complex, LUC) of the honeybee AOTU form two distinct microglomerular synaptic clusters in the lateral bulb (LBU) and medial bulb (MBU) of the LX, respectively (Zeller et al., 2015). These synaptic structures have been described in other insects such as the fruit fly *Drosophila melanogaster* (Hanesch et al., 1989; Seelig and Jayaraman, 2013), the moth *Manduca sexta* (Homberg et al., 1990) and the locust *Schistocerca gregaria* (Träger et al., 2008), but their organization and ultrastructure have never been described in detail in the honeybee. In the locust, where the most precise description of these synaptic microglomeruli has been achieved (Träger et al., 2008), each microglomerulus consists of an extremely large pre-synaptic profile of calycal shape that encompasses numerous post-synaptic profiles of GABAergic tangential neurons of the central body (CB).

The CB is the most prominent neuropil of the central complex (CX), a group of modular structures in the middle brain of insects, which is involved in locomotor control, spatial orientation and visual memory (Pfeiffer and Homberg, 2014). This neuropil is highly connected with the adjacent LX (LAL and BU), which is mainly considered as an important center for processing of sky-compass information. Indeed, in the locust brain, neurons connecting the LAL and the BU (LBU and MBU) to the CX participate in the processing of sky compass signals (Pfeiffer and Homberg, 2014). Additionally, in different insect species, neurons of the LAL convey information from the CX to the thoracic motor centers, thus playing an important role in locomotor control (Namiki and Kanzaki, 2016).

Here we investigated the neural architecture of the microglomerular synaptic clusters in the LBU and MBU of the honeybee brain and provide 3D reconstructions of these structures based on confocal microscopy, as well as ultrastructural and immunohistochemical analyses. We discuss the possible role of these structures in higher-order visual computations achieved at the level of the CX and relate these hypotheses to the visual biology of the honeybee.

## Materials and Methods

### Animals

Free-flying worker honeybee foragers (*Apis mellifera*) were caught at the entrance of an outdoor hive. Bees were placed in small glass vials and cooled on ice until they ceased moving.

### Dextran-Injected Whole Mount Brains

**Figure 1:**
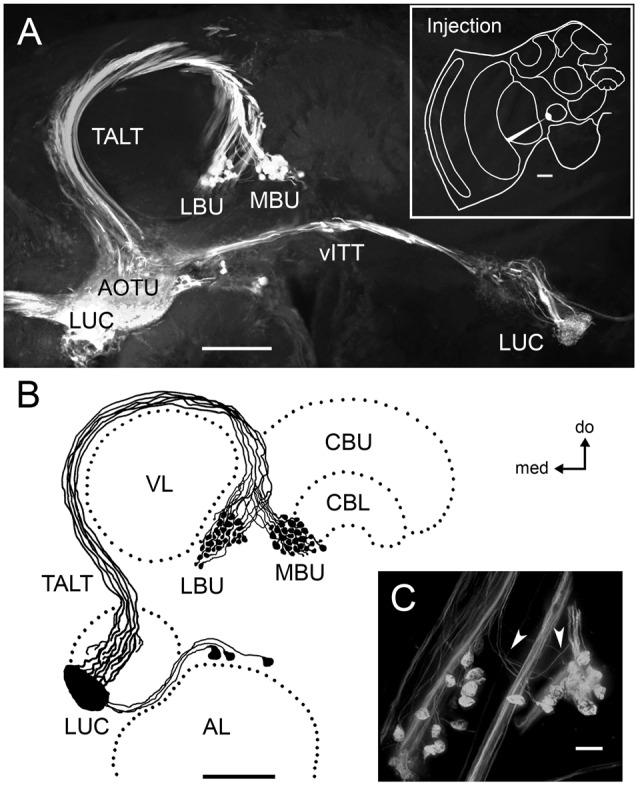
**Neurons connecting the LUC of the AOTU to the LX of the honeybee form large pre-synaptic axon terminals in two microglomerular synaptic clusters. (A)** Dextran tracer injection into the LUC reveals neurons of the TALT running laterally and turning around the ipsilateral VL before innervating the LBU and the MBU of the LX with large glomerular axon terminals. These serial confocal sections of the protocerebrum also show neurons of the vITT connecting the LUC of both brain hemispheres. The upper-right box illustrates the injection site. **(B)** Reconstruction of neural projections connecting the LUC to the LBU and MBU in the mass-fill preparation shown in **(A)**. Cell bodies of these neurons were visible dorsal to the AL and medial to the AOTU. **(C)** Magnification of the microglomerular synaptic clusters in the LX (photograph from Mota et al., 2011). White arrows indicate very thin axon collaterals supplying the pre-synaptic microglomerular profiles. Abbreviations: AL, antennal lobe; AOTU, anterior optic tubercle; CBU, central body upper division; CBL, central body lower division; LBU, lateral bulb; LUC, lower unit complex; LX, lateral complex; MBU, medial bulb; TALT, tubercle-accessory lobe tract; vITT, ventral inter-tubercle tract; VL, vertical lobe; do, dorsal; med, medial. Scale bars: **A,B** = 100 μm; **C** = 10 μm.

**Figure 2:**
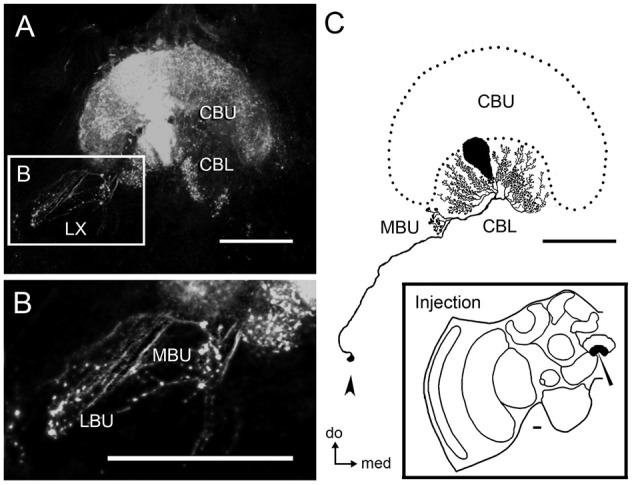
**Tangential neurons connect the LBU and MBU to the CBL. (A)** Serial confocal sections showing neural projections stained by dextran tracer injection into the CBL. This mass-fill preparation reveals wide arborizations in the CBL and CBU, as well as ramifications with blebby structures in the LX. **(B)** Magnification of the LX showing blebby structures in the LBU and MBU. **(C)** Reconstruction of a single tangential neuron from the preparation shown in **(A)** reveals prominent blebby structures confined to the MBU and wide ramifications with small varicosities in different columns of the CBL. The cell body of this neuron, which is most likely post-synaptic, is located in the ventro-posterior portion of the LX (arrow). The lower-right box illustrates the injection site. Abbreviations as in Figure 1. Scale bars = 50 μm.

Bees were individually harnessed in small plastic tubes using low-temperature melting wax to impede the movements of the head. The antennae were fixed frontally using n-eicosan (Aldrich). The head capsule was then opened frontally, salivary glands and a small part of the tracheal sheath were removed, and the brain surface was exposed. Dextran labeled with Texas Red (3000 kDa, Invitrogen) or tetramethyl-rhodamine (10,000 kD, Invitrogen) were used for retrograde or anterograde staining of specific neural pathways. For tracer application in the LUC (Figure 1) of the AOTU, a borosilicate thin-walled glass capillary with the tip covered by a small amount of fluorescent dextran was inserted into this brain region by help of a micromanipulator, and immediately removed after dye injection. For tracer application in the lower division of the central body (CBL, Figure 2), a volume of 0.5 nL of dye solution (10% in 0.1 M sodium phosphate buffer, pH 7.3) was injected into this brain region using a pulled glass capillary (GC 100–10, Harvard Apparatus, Les Ulis, France) connected to a pressure microinjector (IM 300, Narishige, London, UK). To achieve specific injection into the CBL, the capillary connected to a micromanipulator was first positioned at the anterior brain surface, 200 μm ventral to the middle point between the basal rings of the two medial calyces of the MBs. The capillary tip was then inserted to a depth of approximately 350 μm from the anterior brain surface before dye injection. After dextran injections, the head capsule was closed, and animals were fed with 50% sucrose solution and kept alive in a moist chamber for approximately 12 h. Tracer-filled brains were then dissected out, fixed in phosphate-buffered paraformaldehyde solution (4%; pH 6.8) for at least 24 h, dehydrated in ascending concentrations of ethanol, and cleared in methyl salicylate (Sigma-Aldrich) for 24 h.

### GABA and 5HT Immunohistochemistry on Whole Mount Brains

For immunohistochemistry in whole mount brains, bees were quickly immobilized on ice and the antennae were cut close to the basal articulation. Bees were perfused with the fixative by injecting 4% paraformaldehyde in PBS (pH 7.4) into the thorax until drops of fixative extruded from the cut ends of the antennae (Kreissl et al., 2010). The brains were then dissected in fixative solution and post-fixed for 2–3 h at room temperature (RT). During this period, fixative solution was changed at least once. Then, brains were repeatedly washed in PBS with 0.2% Triton X-100 (PBS-TX 0.2) during 12 h. All incubations and washing steps were done with constant gentle agitation. Brains were pre-incubated at 4^o^C in blocking buffer (PBS containing 0.5% Triton X-100, 0.2% BSA and 0.02% NaN_3_) overnight. Anti-GABA or anti-5HT antisera produced in rabbit were applied at 1:3000 and 1:1000, respectively. We used anti GABA 4TB, kindly provided by Dr. H. Dircksen (University of Stockholm), which has been characterized and used in bees previously (Homberg et al., 1999; Kreissl et al., 2010). For 5HT staining we used anti-serotonin S5545 (SIGMA Aldrich, Munich). Each brain was incubated for 6–7 days at RT in 1 ml of solution containing the primary antisera diluted in PBS-TX 0.2 with 0.2% BSA and 0.02% NaN_3_. After incubation with the primary antisera, the brains were washed with PBS-TX 0.2 for 10–12 times 30 min at RT. Secondary F(ab′)2 fragments of goat anti-rabbit antibodies conjugated to Alexa 488 (Invitrogen, Carlsbad, CA, USA) were used at 1:500 in PBS-TX 0.2 with 0.2% BSA and 0.02% NaN_3_ for 6–7 days at RT. Subsequently, the brains were washed repeatedly in PBS-TX 0.2, dehydrated in an ethanol series (50%, 70%, 90%, 98%, 100%, 100%, 30 min each), cleared in xylene (2 times for 5 min), and mounted in DPX (SIGMA-Aldrich, Munich, Germany) between two coverslips spaced by custom-made metal frames of 0.6 mm thickness.

### 5HT/Synapsin Double Staining

In another set of experiments, we co-stained anti-5HT and monoclonal anti-synapsin I antibodies to analyze if anti-5HT reactive arborizations were indeed connected to microglomeruli in the LBU and MBU. Brains were fixed in 4% paraformaldehyde in PBS (pH 7.4) for 12 h, dissected in PBS, embedded in 5% low melting point agarose (Agarose II, no. 210–815, AMRESCO) and sectioned in a frontal plane (150 μm) with a vibrating microtome (Leica VT 1000S). Free-floating sections were pre-incubated in PBS-TX 0.2 and 2% normal goat serum for 2 h, and then incubated with anti-5HT antibodies (as described above) for 2 h at RT. After extensive washing in PBS-TX 0.2 for 2 h, sections were incubated with a monoclonal anti-synapsin I antibody (1:50; SYNORF1; Developmental Studies Hybridoma Bank, University of Iowa, Iowa City, IA, USA) for 2 h at RT. After incubation with the primary antisera, preparations were repeatedly washed (4 times for 30 min) in PBS-TX 0.2 for 2 h and incubated in the secondary antibody Alexa 488-conjugated goat anti-rabbit (Invitrogen: 1:500) in PBS with 1% normal goat serum for 2 h at RT. Brain sections were then repeatedly washed (4 times for 15 min) in PBS-TX 0.2 for 1 h at RT, and incubated in the secondary antibody Alexa 546-conjugated goat anti-mouse (Invitrogen: 1:250) in PBS with 1% normal goat serum for 2 h at RT. Brains sections were washed (4 times for 15 min) in PBS-TX 0.2 and mounted on coverslips with Vectashield medium (VectorLab, France).

### Synapsin/Phalloidin Double Staining

Microglomeruli were labeled and quantified adapting a published protocol for double staining pre-synaptic and post-synaptic profiles (Groh et al., 2006; Krofczik et al., 2008; Hourcade et al., 2010). Brains were embedded in 5% low melting point agarose (Agarose II, no. 210–815, AMRESCO) and sectioned in a frontal plane (200 μm) with a vibrating microtome (Leica VT 1000S). Free-floating sections were repeatedly washed (3 times for 10 min) in PBS with 2% Triton X-100 and pre-incubated in PBS with 0.2% Triton X-100 and 2% normal goat serum for 1 h at RT. Preparations were then incubated for 4 days at 4^o^C simultaneously in 0.2 U of Alexa Fluor 488 phalloidin (Invitrogen, A-12379) and a monoclonal anti-synapsin I antibody (1:50; SYNORF1; Developmental Studies Hybridoma Bank, University of Iowa, Iowa City, IA, USA). After repeated washes (5 times for 10 min) in PBS, preparations were incubated in the secondary antibody (Alexa Fluor 546-conjugated goat anti-mouse, Invitrogen: 1:250 in PBS with 1% normal goat serum) for 2 h at RT. Brains sections were washed (5 times for 10 min) in PBS, transferred to 50% glycerol in PBS for 15 min and mounted on coverslips with 80% glycerol/PBS solution.

### Confocal Analysis and Image Processing

Images of dextran-injected whole mount brains, as well as double-stained 5HT/synapsin and synapsin/phalloidin immunopreparations were taken with a Leica TCS SP5 confocal laser scanning microscope (Leica Microsystems) using DPSS 561 nm and/or Argon laser. Depending on the required magnification and resolution, we used either a 10× air objective (Plan-Fluotar, 0.3 NA, Leica) or a 20× water immersion objective (HC Plan Apo, 0.7 NA, Leica) or a 63× oil immersion (Plan-Apo, 1.4 NA, Leica). Image stacks were acquired with optical slice thickness of 11 μm (10× objective) or 1.7 μm (20× objective) or 0.7 μm (63× objective) with pinhole size of 1 Airy unit and resolution of 1024 × 1024 pixels per frame. Excitation and emission wavelengths of the chromophores used were respectively 596 nm/615 nm (Texas Red), 557 nm/576 nm (tetramethyl-rhodamine), 561 nm/570–620 nm (Alexa 546) and 488 nm/500–550 nm (Alexa 488). Projections of confocal stacks containing mass-filling or immunoreactive neuronal staining were achieved using ImageJ (Rasband, National Institutes of Health, Bethesda, MD, USA). For immunohistochemical double staining, the two channels were merged using pseudocolors in ImageJ software. Image brightness and contrast were adjusted using Adobe Photoshop CS5 (Adobe Systems, San Jose, CA, USA). Reconstructions of neural populations or single neurons (Figures 1, 2, respectively) were achieved by drawing over frontal stacks of serial aligned micrographs imported to Adobe Photoshop CS5.

Images of GABA and 5HT immunostaining on whole mount brains were acquired with a Zeiss LSM 510 NLO confocal laser scanning microscope (Carl Zeiss, Inc., Thornwood, NY, USA) using Argon laser. We used either a 10× (Plan-Apo, 0.45 NA, Zeiss) or a 20× (W Plan-Apo, 1.0 NA, Zeiss) water immersion objective. Image stacks were acquired with optical slice thickness of 6 μm (10× objective) or 0.7 μm (20× objective) with pinhole size of 1 Airy unit and resolution of 1024 × 1024 pixels per frame. Excitation and emission wavelengths of the Alexa 488 chromophore conjugated to the second antibodies were 488 nm/500–550 nm, respectively. Projections of confocal stacks containing immunoreactive neuronal staining were achieved using Zeiss LSM image browser (Carl Zeiss, Inc., Thornwood, NY, USA). Image brightness and contrast were adjusted using Adobe Photoshop CS4 (Adobe Systems, San Jose, CA, USA).

### 3D Reconstructions, Glomerular Counting and Volume Measurements

Whole confocal stacks of 1024 × 1024 pixels (scanned with a 20× objective) obtained from 10 brains with high-contrast double staining of the pre- and post-synaptic microglomerular profiles were selected for three-dimensional reconstruction in AMIRA 5.3.2 (Mercury Computer Systems, Chelmsford, MA, USA). For reconstruction of entire microglomerular cluster volumes, both pre- and post-synaptic profiles were bounded slice-by-slice using the threshold segmentation method (Figures 7, 8). We refined threshold segmentation of specific staining contours by applying smoothing operation (*Smooth Labels)*. The two channels (pre-synaptic and post-synaptic specific staining) were treated separately during threshold segmentation and were merged during volume rendering. This method required less manual interaction during reconstruction and allowed a high fidelity of the 3D reconstruction to the original double immunostaining (Figures 7, 8). The number of microglomeruli in each microglomerular cluster was estimated using the pre-synaptic staining (anti-synapsin). Reconstruction of individual glomeruli was achieved by manually outlining the contours of pre- and post-synaptic profiles on each section, interpolating the label between sections, and then performing surface rendering (Figure 9). The software provided a volume estimate of each material reconstructed from the serial surfaces.

### Transmission Electron Microscopy (TEM) and Ultrastructural Analysis

The brains were dissected and subsequently fixed with 2% paraformaldehyde/2.5% glutaraldehyde in Sorensen’s buffer (0.1 M, pH 7.2) overnight at 4°C. They were washed three times in Sorensen’s buffer and post-fixed in 1% OsO_4_ for 1 h at RT. After several rinses in the same buffer, the samples were dehydrated in a graded series of ethanol and embedded in epoxy resin. Then, we cut ultra-thin frontal sections (80 nm, Leica Ultracut microtome). We mounted the slices on formvar-carbon-coated grids and counterstained with 1% aqueous uranyl acetate and lead citrate. The grids were finally examined at 80 kV on a Jeol JEM-1400 electron microscope. Images were acquired using a CCD camera (Gatan, Orius SC1000b) at different magnifications. We used the *Fit Ellipse* command and the *Shape Descriptors* application of ImageJ (Wayne Rasband, National Institutes of Health, Bethesda, MD, USA) to calculate the minor (*Mi*) and major (*Ma*) ellipsoid axes of glomerular sections in the LBU and MBU, as well as the total ellipsoid area (*A = π* ×* Mi*/2 ×* Ma*/2) and a roundness index (*R = Mi/Ma*). When the value of *R* is 1.0, shape is perfectly circular. As the values approach 0.0, it indicates an increasingly elongated shape.

### Statistical Analysis

We used one-way ANOVA to compare the values of minor ellipsoid axis (*Mi*), major (*Ma*) ellipsoid axis, total ellipsoid area (*A*) and roundness index (*R*) in glomerular ultra-sections of the lateral and medial glomerular cluster. One-way ANOVA was also used to compare the distance between the outer and inner membranes of the pre-synaptic profiles and the ratio of pre-synaptic to post-synaptic volume measured in individual LBU and MBU microglomeruli. Two-way ANOVA followed by Tukey’s test was used for comparing the following: (i) number of synaptic microglomeruli in the LBU and MBU from the right and left brain hemispheres of 10 bees (Figure 7); (ii) pre- and post-synaptic volumes in both LBU and MBU microglomerular clusters of 10 bees (Figure 8); (iii) pre- and post-synaptic volumes in 10 individual glomeruli randomly selected in a LBU and a MBU cluster of 10 bees (Figure 9). All values are given as mean and standard deviation.

### Axes and Nomenclature

Brain structures are described following insect brain nomenclature conventions proposed by Ito et al. (2014). Positional information is described according to the body axis (not the neuraxis), in which the MB calyces are dorsal, the antennal lobes (AL) ventral and anterior, and the subesophageal ganglion ventral and posterior (Ito et al., 2014).

## Results

We analyzed the neural organization and ultrastructure of conspicuous microglomerular synaptic clusters in the LBU and MBU of the honeybee LX using different neuroanatomical and immunohistochemical methods. Pre-synaptic microglomerular profiles are composed of large axon terminals of TALT neurons (Mota et al., 2011) conveying information from the LUC (Zeller et al., 2015) of the AOTU to the LX (Figure 1). Post-synaptic microglomerular profiles involve assemblies of small bleb-like dendritic specializations of tangential neurons providing input from the LX to the CBL in the middle brain (Figure 2). Below we describe the neural architecture, the pre- and post-synaptic arrangement and the neurotransmitters that we could identify in these microglomerular synaptic clusters.

### Neural Connectivity of Microglomerular Synaptic Clusters in the LX

Localized mass filling dextran injections in the LUC revealed pre-synaptic neurons running through the TALT that form large knob-shaped axon terminals in the LX (Figure 1A). These terminals are segregated in two distinct clusters of synaptic microglomeruli: one at the LBU and the other at the MBU. Cell bodies of these neurons are located dorsally to the AL, in the medial vicinity of the AOTU (Figure 1B). Very thin axon collaterals from TALT neurons supply the large microglomerular knob-shaped axon terminals in the LBU and MBU (Figure 1C).

Injections of neuronal tracer in the CBL revealed tangential neurons with varicosities in the LBU and MBU (Figures 2A,B). Cell bodies of these neurons are located in the ventralmost portion of the LX, dorsal to the AL (Figure 2C). Figure 2C shows one of these CBL tangential neurons, which was traced entirely from a mass filling preparation. The primary neurite of this neuron runs via the isthmus tract and emits a collateral projection with varicosities in the MBU, as well as wide fan-shaped arborizations with small bleb-like varicosities in different columnar layers of the CBL.

### GABA and 5HT Immunohistochemistry in the Microglomeruli

**Figure 3:**
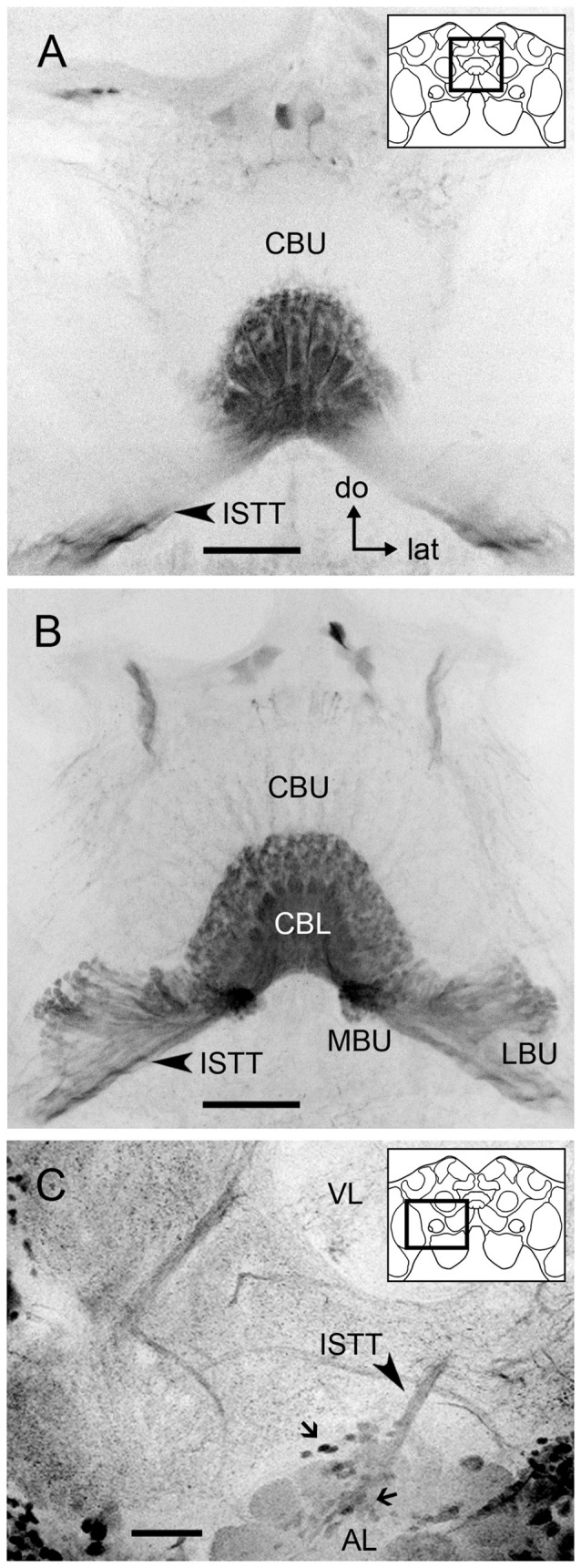
**GABA immunoreactivity in tangential neurons connecting the CBL to the microglomerular synaptic clusters. (A)** Frontal optical section of the medial protocerebrum showing GABA immunoreactive projections from the isthmus tract (ISTT, arrow) providing dense fan-shaped arborizations in all columnar layers of the CBL. These arborizations contain numerous small bleb-like structures. The upper-right sketch indicates the brain area shown in **(A,B)**. **(B)** Serial frontal sections following posteriorly the one in **(A)** show that the dense and wide arborizations in the CBL are connected to prominent varicosities that are packed in the MBU and LBU. Few arborizations are visible in the CBU. **(C)** Stack of the right anterior lateral protocerebrum (60 μm depth) showing a group of GABA immunoreactive somata (small arrows) dorsal to the antennal lobe and giving rise to the ISTT that connects to the GABAergic neural processes in the CB **(A,B)** and the microglomerular clusters in the MBU and LBU **(B)**. The upper-right sketch indicates the brain area shown in **(C)**. Abbreviations as in Figure 1. (do = dorsal, lat = lateral). Scale bar: 50 μm.

We identified numerous GABA-immunoreactive tangential neurons connecting the microglomerular synaptic clusters in the LBU and MBU to the CBL (Figures 3A,B). These neurons present dense fan-shaped arborizations in all columnar layers of the CBL (Figures 3A,B) and numerous varicosities in the microglomerular structures of the LBU and MBU (Figure 3B). Arborizations in the dorsal portion of the CBL usually comprise numerous bleb-like varicosities (Figures 3A,B). Very few GABA-immunoreactive arborizations are visible in the CBU (Figure 3B). A prominent connection is observed between these tangential neurons and a group of GABAergic somata just dorsal to the AL (Figure 3C). We counted 219–228 GABAergic somata per brain hemisphere in this group, whose primary neurites give rise to the isthmus tract (Figure 3C). Considering the general morphology described above, the individual neuron traced in Figure 2 is probably one of these GABAergic tangential neurons.

**Figure 4:**
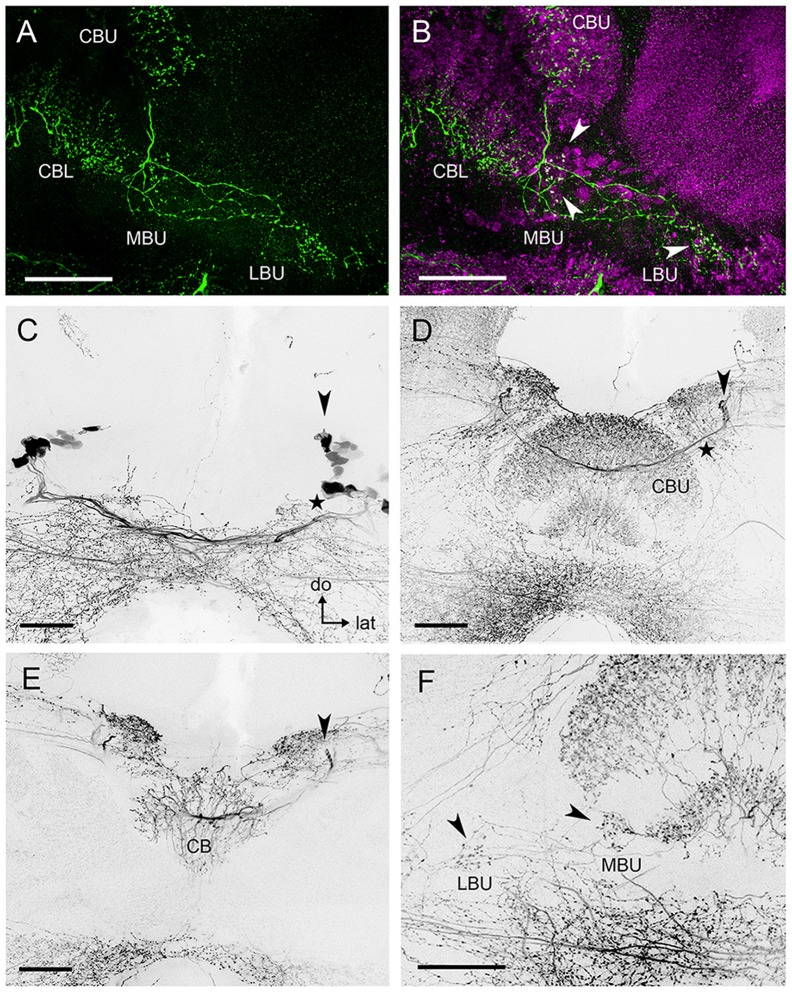
**Serotonin-immunoreactive neurons connecting to the microglomerular synaptic clusters present wide arborizations in the CBL and CBU. (A)** Neuronal processes with small varicosities in the LBU and MBU are revealed by anti-5HT staining. **(B)** Double synapsin/5HT staining shows that some of the serotonergic varicosities (green) are co-localized (arrows) with pre-synaptic staining (magenta) in the microglomerular synaptic clusters of the LBU and MBU. **(C)** Some serotonin-reactive somata in the posterior superior protocerebrum (arrow) are separated from a second group of somata lying slightly more ventrally (star). The latter contribute to a prominent bundle of neurites that is posterior to the CB (star) and does not innervate the CBU and the CBL. **(D)** The primary neurites (arrow) of the dorsal somata shown in **(C)** send primary neurites to the anterior surface of the CBU and continue towards the contralateral side of the brain. They form a common interhemispheric bundle (star) with the primary neurites of their contralateral equivalents. **(E)** Substack of **(D)** showing collaterals of the interhemispheric bundle invading the CB. These collaterals give rise to the wide immunoreactive arborizations observed in the CBU and CBL, which are visible in **(D)**. Primary neurites of the dorsal somata are indicated by the arrow, as in **(D)**. **(F)** Substack of **(D)** showing serotonin immunoreactive neurites connecting the CBU and CBL with the MBU and LBU. Arrows indicate varicosities corresponding to those shown in **(A)**, which can be associated to the microglomerular clusters **(B)**. Abbreviations as in Figures 1–3. Scale bars: 50 μm.

Serotonin-immunoreactive neuronal processes with small varicosities were also identified in the LBU and MBU (Figure 4A). In order to analyze if microglomeruli from the LBU and MBU are indeed connected to these serotonin-reactive varicosities, we performed double anti-synapsin/anti-serotonin staining. We found that part of the pre-synaptic LBU and MBU microglomerular profiles revealed by anti-synapsin staining were co-localized with small varicosities of serotonergic neurons (Figure 4B). This result suggests that these small varicosities are pre-synaptic. Anti-synapsin/anti-serotonin colocalization was more frequent in MBU microglomeruli than in LBU microglomeruli, while most of the serotonergic varicosities do not co-localize with pre-synaptic microglomerular structures (Figure 4B). We counted a maximum of 21 MBU microglomeruli and eight LBU microglomeruli (in four preparations) presenting such co-localization.

The neurons giving rise to the serotonin-immunoreactive processes in the LX and the CB appear to be broad field neurons with a soma in the group 4 (Figure 4C) of posterior serotonergic neurons (Schürmann and Klemm, 1984). In each hemisphere, at least two serotonin-immunoreactive somata (arrow in Figure 4C) in the posterior cluster have wide-ranging projections. Their primary neurites project first dorsally, then turn anteriorly, pass over the CBU and then turn towards the contralateral side of the brain (Figure 4D). Just in front of the CBU, they form a common bundle with the primary neurites of their contralateral partners (Figure 4D). From this bundle, they give off collaterals which project posteriorly and invade the CBU and CBL (Figure 4E). In the CBU, as well as in the CBL, many projections with varicosities are arranged in a columnar manner (Figures 4D,F). In the MBU and LBU, neuronal processes give rise to small varicosities (Figure 4F) that are distributed in the vicinity of the microglomerular synaptic clusters or directly connected to their pre-synaptic elements (Figure 4B). Other collaterals project from the common bundle anteriorly to invade anterior brain areas. The main branches continue towards the contralateral anterior superior protocerebrum above the MB lobes. The synaptic polarity of these neurons was difficult to define and we could not further trace the neurons in the dense meshwork of serotonin immunoreactive neurites in other regions of the brain.

### Microglomerular Ultrastructure in the LBU and MBU

**Figure 5:**
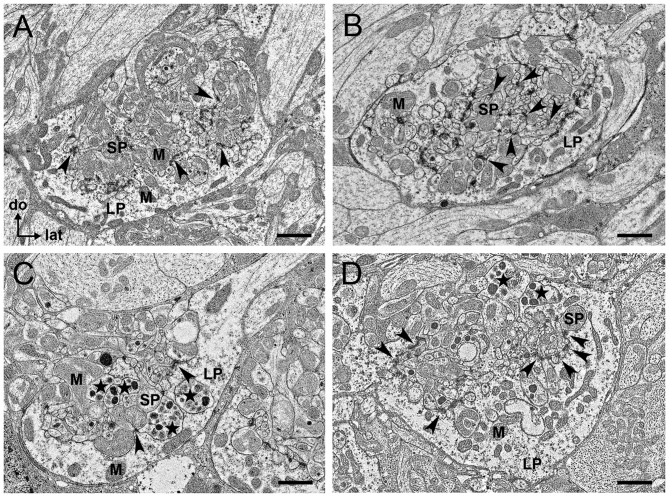
**Transmission electron microscopy (TEM) sections of individual microglomeruli in the LBU (A,B) and MBU (C,D).** In all these four examples of microglomerular ultrastructure **(A–D)**, the pre-synaptic profile is characterized by a large profile (LP) enclosing numerous post-synaptic small profiles (SPs). Copious small vesicles of different electron densities are visible in the LP. Both the LP and the SP have numerous mitochondria (M). Black arrowheads indicate synaptic contacts. **(A)** Many synaptic contacts are visible between the LP and SP in a microglomerulus of the LBU. **(B)** Apart from the numerous synaptic contacts between LP and SP, synaptic contacts between different post-synaptic SP (arrowheads) seem to exist in this microglomerulus of the LBU. These synapses between SP were not visible in microglomeruli of the MBU **(C,D)**. In MBU microglomeruli **(C,D)**, some of the SP enclose very large and electron-dense vesicles (stars) that were not visible in LBU microglomeruli **(A,B)**. Abbreviations as in Figures 1–3. Scale bars: 1 μm.

TEM revealed similarities and differences in the ultrastructure and cellular composition of microglomeruli in the LBU (Figures 5A,B) and MBU (Figures 5C,D). Microglomeruli in these synaptic clusters are typically composed of a large pre-synaptic cup-shaped outer profile that embraces numerous small post-synaptic inner profiles. The large pre-synaptic profile is bounded by a glial sheath and comprises many small clear vesicles, as well as dense-core vesicles. Numerous mitochondria are present both in the large pre-synaptic profile and in the small post-synaptic profiles (Figure 5). In some of the MBU microglomeruli, we also identified profiles that enclose very large (from 70 nm up to 200 nm) dense-core vesicles (Figures 5C,D), which were not observed in LBU microglomeruli (Figures 5A,B). We could not clearly determine the synaptic polarity of these profiles, but the presence of these large vesicles suggest they are pre-synaptic.

We measured the size of the minor and the major axis in frontal ellipsoid sections of the LBU and MBU microglomeruli, and used these values to calculate the area and the roundness of glomerular sections. Minor ellipsoid axes in glomeruli of the MBU (*N* = 147 sections from six brains) vary from 3.8 μm to 9.9 μm (7.3 ± 2.8 μm), whereas major ellipsoid axes vary from 4.7 μm to 11.9 μm (8.4 ± 3.5 μm). In the LBU (*N* = 147 sections from six brains), we found minor ellipsoid axes varying from 3.2 μm to 8.3 μm (5.3 ± 2.6 μm) and major axes varying from 4.4 μm to 11.3 μm (8.2 ± 3.1 μm). Total ellipsoid areas (MBU: 50.7 ± 19.6 μm^2^; LBU: 39.2 ± 16.7 μm^2^), as well as roundness indexes (MBU: 0.71 ± 0.12; LBU: 0.64 ± 0.15), are significantly higher in the MBU than in the LBU glomerular sections (*ellipsoid area × microglomerular cluster* ANOVA, *F*_(1,292)_ = 63.3, *p* < 0.001; *roundness × microglomerular cluster* ANOVA, *F*_(1,292)_ = 122.6, *p* < 0.001).

Pre-synaptic profiles in the MBU (Figures 5C,D) appear to be larger than the pre-synaptic profiles of the LBU (Figures 5A,B). To quantify this feature, we measured the largest distance between the outer and the inner membrane of pre-synaptic profile sections (*N* = 147 sections from six brains per microglomerular cluster) and we found that these values are significantly higher in the MBU than in the LBU (MBU: 3.2 ± 1.1 μm; LBU: 2.5 ± 0.8 μm; *distance × microglomerular cluster* ANOVA, *F*_(1,292)_ = 40.9, *p* < 0.001).

Several synaptic contacts could be observed at the inner membrane of the large cup-shaped profile that encloses the post-synaptic profiles (Figure 5). We also identified synaptic contacts between post-synaptic profiles of few LBU glomeruli (Figure 5B), but these contacts were not observed in the MBU glomerular sections analyzed. All synaptic contacts present the typical ultrastructural aspect of chemical synapses: an electron-dense pre-synaptic bar surrounded by vesicle assemblies, as well as electron-dense material around the inner membrane of the post-synaptic profile (Strausfeld, 1976; Träger et al., 2008).

### Double Staining and Glomerular Counting

**Figure 6:**
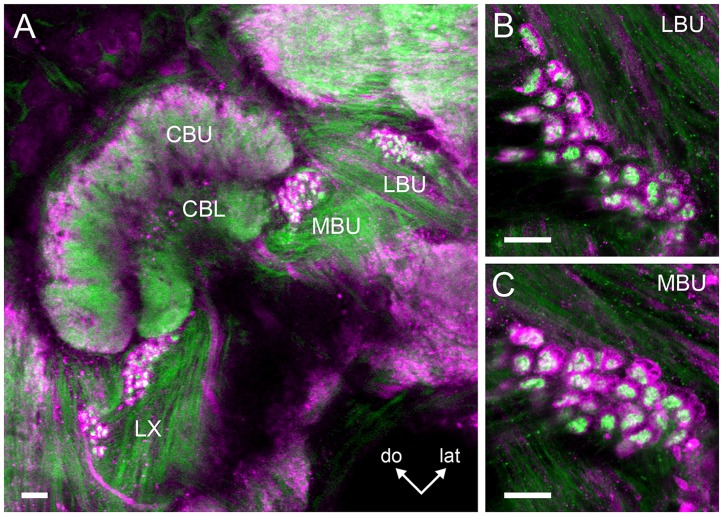
**Double staining of the pre- and post-synaptic profiles in the microglomerular clusters of the LBU and MBU. (A)** Serial confocal sections (100 μm depth) show prominent pre-synaptic profiles reactive to anti-synapsin antibody (in magenta) enclosing blebby post-synaptic profiles reactive to anti-phalloidin antibody (in green). Magnifications of the synaptic microglomeruli in the LBU and MBU are shown in **(B,C)**, respectively. In both microglomerular clusters, pre-synaptic profiles have a cup-shaped structure (in magenta) that embrace numerous blebby post-synaptic profiles (in green). Abbreviations as in Figures 1–3. Scale bars: **A** = 50 μm; **B,C** = 10 μm.

**Figure 7:**
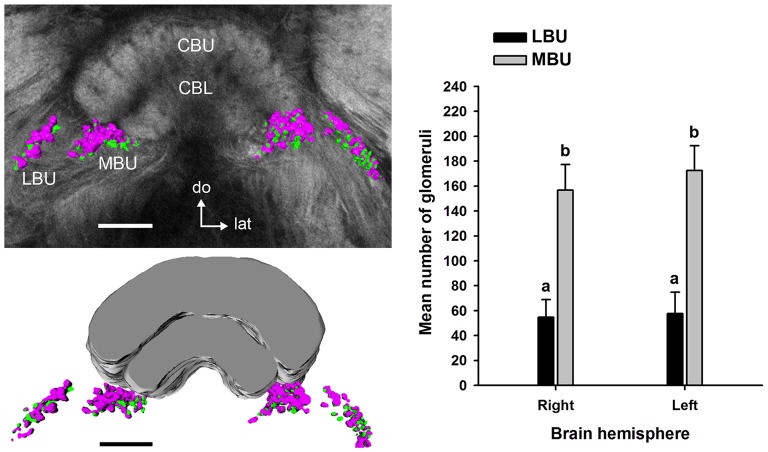
**Mean number of microglomeruli in the LBU and MBU of both honeybee brain hemispheres (*N* = 10 brains).** Left panels: three-dimensional rendering of the pre-synaptic (in magenta) and post-synaptic (in green) microglomerular structures from frontal serial confocal sections of a double staining preparation (shown in Figure 6). The number of microglomeruli was calculated in the LBU and MBU of the right and left brain hemispheres as the number of pre-synaptic profiles (anti-synapsin reactive, see Figure 6). The mean number of microglomeruli in the MBU is significantly higher than in the LBU of both brain hemispheres. Different small letters on top of bars indicate significant differences (*p* < 0.05; two-way ANOVA). No difference was found between the numbers of LBU and MBU microglomeruli of the right and the left brain hemisphere. Abbreviations as in Figures 1–3. Scale bars: 100 μm.

Double synapsin/phalloidin staining of pre- and post-synaptic profiles revealed that microglomeruli in the two synaptic clusters in the LX are tightly packed (LBU and MBU; Figure 6). These clusters are situated between approximately 300 μm and 480 μm beneath the frontal brain surface (measured in non-dehydrated specimen). We found an important difference in the number of synaptic microglomeruli between the LBU and the MBU in both the brain hemispheres (*N* = 10 brains; Right hemisphere: LBU = 55 ± 14, MBU = 157 ± 21; Left hemisphere: LBU = 58 ± 18, MBU = 173 ± 20; Figure 7). A *number of glomeruli × brain hemisphere × microglomerular cluster* ANOVA showed that the MBU has significantly more microglomeruli than the LBU (Factor *microglomerular cluster*: *F*_(1,36)_ = 356.5; *p* < 0.00001), but there was no difference between the two brain hemispheres (Factor *brain hemisphere*: *F*_(1,36)_ = 1.3; NS).

### Arrangement of the Pre- and Post-synaptic Glomerular Volumes

**Figure 8:**
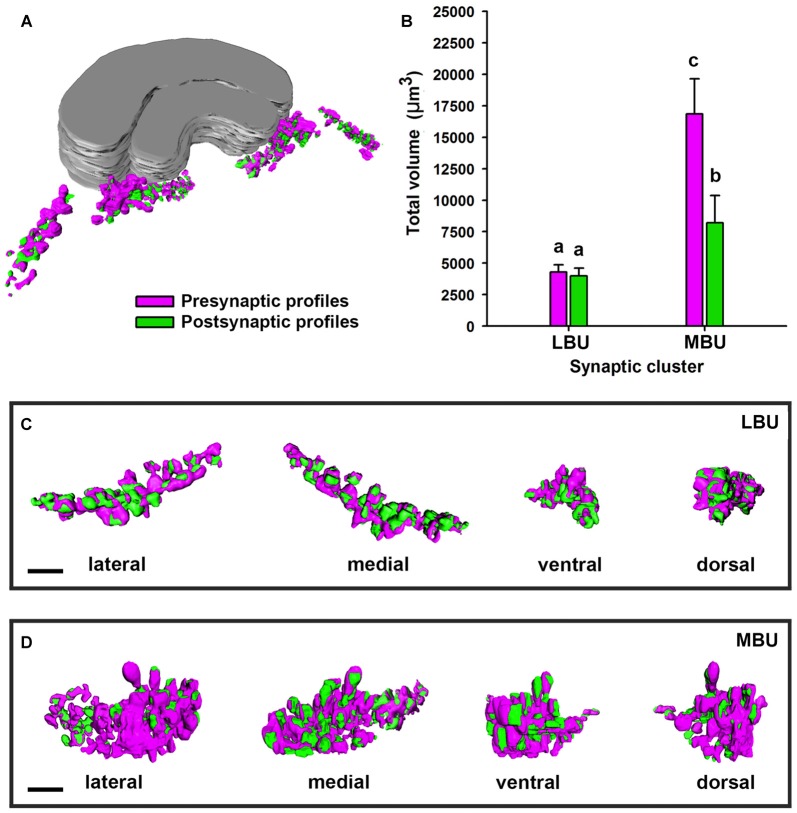
**Three-dimensional structure and volume measurements of the pre-synaptic and post-synaptic profiles in the LBU and MBU of the honeybee LX.** **(A)** Image shows a reconstruction of the pre-synaptic (in magenta) and post-synaptic (in green) profiles in a whole brain. **(B)** Mean total volume of the pre-synaptic and post-synaptic profiles measured from 3D reconstructions of an entire LBU and MBU from a single brain hemisphere (*N* = 10 brains). The total volume of pre- and post-synaptic profiles in the LBU microglomerular cluster is similar, while it was higher for the pre-synaptic than for the post-synaptic profiles of the MBU. Boxes show lateral, medial, ventral and dorsal views of a LBU **(C)** and a MBU **(D)** microglomerular synaptic structure. Different small letters on top of bars indicate significant differences (*p* < 0.05; two-way ANOVA). Scale bars = 10 μm.

**Figure 9:**
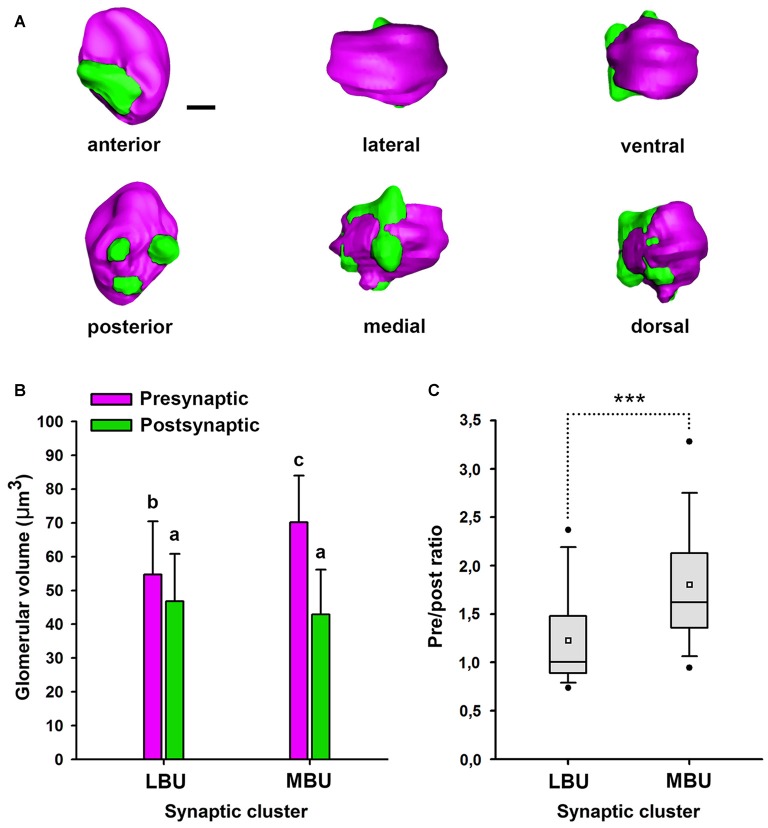
**Three-dimensional structure and volume measurements of the pre-synaptic and post-synaptic profiles in individual glomeruli of the LBU and MBU.** **(A)** Anterior, posterior, lateral, medial, ventral and dorsal views of an individual microglomerulus of the right LBU. Scale bar = 1 μm. **(B)** Mean volume of the pre- and post-synaptic profiles measured in individual microglomeruli of the LBU and MBU microglomerular clusters (*N* = 100 microglomeruli/cluster, randomly selected in 10 brains). Both in LBU and MBU individual microglomeruli, the pre-synaptic volume is significantly larger than the post-synaptic volume. The volume of the pre-synaptic profile of individual MBU glomeruli is significantly larger than that of the LBU glomeruli, while the volumes of post-synaptic profiles did not differ between LBU and MBU. Different small letters on top of bars indicate significant differences (*p* < 0.05; two-way ANOVA). **(C)** Box and whisker plots of the ratio of pre-synaptic to post-synaptic volume measured in individual LBU and MBU microglomeruli (*N* = 100 microglomeruli/cluster, randomly selected in 10 brains). The horizontal line within the box shows the median. The white square inside the box shows the mean. The boundaries of the box indicate the 25th and 75th percentile. Whiskers indicate the minimum and maximum values. Lower and upper black dots show the 5th and the 95th percentile (*outliers*). Asterisks indicate significant differences (*p* < 0.0001; one-way ANOVA).

We performed 3D reconstructions of the microglomerular synaptic clusters in the LBU and MBU (Figure 8) and we only found significant differences between their pre- and post-synaptic volumes in the MBU (Figure 8; *two way ANOVA*: factor *LBU/MBU F*_(1,36)_ = 213.6, *p* < 0.00001; factor *pre/post F*_(1,36)_ = 61.2, *p* < 0.0001; interaction *F*_(1,36)_ = 53.1, *p* < 0.0001). The pre-synaptic volume is significantly higher than the post-synaptic volume in the MBU microglomerular cluster (Figure 8; Tukey test, *p* < 0.001), but not in the LBU microglomerular cluster (Figure 8; Tukey test, NS). Besides, both the pre-synaptic and the post-synaptic volumes of the MBU microglomerular cluster are significantly higher than the ones of the LBU microglomerular cluster (Figure 8; Tukey test, *p* < 0.001 in both cases). To analyze if the differences in the pre- and post-synaptic volumes measured in the whole LBU and MBU microglomerular structures (Figure 8) are simply due to differences in the number of glomeruli (Figure 7) or are due to differences at the level of individual glomeruli, we reconstructed 10 individual LBU and MBU microglomeruli randomly selected in 10 brains (*N* = 100 glomeruli per cluster; Figures 9A,B). The pre-synaptic volume exceeds the post-synaptic volume of individual glomeruli in both microglomerular clusters (Figure 9B; *volume × synaptic profiles × microglomerular cluster* ANOVA, Interaction *microglomerular cluster × synaptic profiles F*_(1,396)_ = 48.3, *p* < 0.0001; Tukey test, *p* < 0.01 for LBU and *p* < 0.0001 for MBU). In addition, pre-synaptic volumes of individual glomeruli in the MBU are significantly higher than pre-synaptic volumes of glomeruli in the LBU (Figure 9B; Tukey test, *p* < 0.001), whereas post-synaptic volumes do not differ significantly between MBU and LBU individual glomeruli (Figure 9B; Tukey test, NS). We then analyzed the ratio of pre-synaptic to post-synaptic volume in individual glomeruli of the LBU and MBU (Figure 9C). The pre/post-synaptic ratios of LBU glomeruli were close to 1.0 (median = 1.0, mean = 1.2), while the ratios of MBU glomeruli were higher than 1.5 (median = 1.6; mean = 1.8). This result indicates that most of the LBU glomeruli have similar pre- and post-synaptic volumes (Figure 9C), although the average value of pre-synaptic volume measured in the LBU is slightly higher than the post-synaptic value (Figure 9B). The pre/post synaptic ratios of the MBU glomeruli are significantly higher than those of the LBU glomeruli (Figure 9C; *pre/post ratio × microglomerular cluster* ANOVA, *F*_(1,198)_ = 40.38, *p* < 0.0001).

## Discussion

We analyzed the synaptic organization of the microglomerular clusters in the LBU and MBU of the honeybee brain by means of neuronal tracing techniques, immunohistochemistry, TEM and three-dimensional reconstructions. We found that the pre-synaptic portion of these microglomerular structures is composed of large cup-shaped profiles originating from axon terminals of TALT neurons (Mota et al., 2011) connecting the AOTU-LUC to the LBU and MBU (Zeller et al., 2015). Small post-synaptic profiles embraced by the large pre-synaptic profile of a microglomerulus are most likely composed of varicose dendritic specializations of numerous GABAergic tangential neurons connecting the LBU and the MBU to the CBL. We also identified serotonergic broad field neurons that might provide modulatory input from the CBL and CBU to their corresponding microglomeruli. Although the ultrastructure of LBU and MBU microglomeruli was very similar, large dense core vesicles were found in some of the MBU microglomeruli that were not found in LBU microglomeruli. Moreover, the number of MBU microglomeruli and the volume of their pre-synaptic profiles are higher than those of LBU microglomeruli.

### Neurotransmission in the Synaptic Microglomeruli of the LBU and MBU

Kreissl and Bicker (1989) found acetylcholinesterase (AchE) activity and acetylcholine receptor-like (AchR) immunoreactivity in the region corresponding to the microglomerular structures described here. At that time, they were confounded with clusters of small cell bodies. A comparison of these findings with our work suggests that the cholinergic activity reported could be located in the large pre-synaptic rather than in the post-synaptic profiles of LBU and MBU microglomeruli (Figures 1, 2).

GABAergic tangential neurons connecting the LBU and MBU to the CBL presumably contribute to most of the small post-synaptic profiles observed at the electron microscopic level (Figure 5). This can be assumed because of the general morphology of the approximately 225 GABA-reactive neurons identified per brain hemisphere (Figure 3). These tangential neurons have profuse varicosities in the LBU and MBU, which are very similar to the ones observed in the post-synaptic microglomerular profiles stained in double synapsin/phalloidin preparations (Figure 6). Besides, the GABAergic lateral projections connecting CBL to the varicosities in the LBU and MBU (Figure 3B) appear as post-synaptic in double synapsin/phalloidin staining (Figure 6). Similarly to Schäfer and Bicker (1986), we found that GABA reactive neurons have very dense arborizations in the CBL, but very few processes in the CBU. Whether these few CBU ramifications are connected to the synaptic microglomeruli in the LBU and MBU still needs to be confirmed. The general aspect of the single neuron traced in Figure 2, which has ramifications restricted to the MBU and the CBL, strongly suggests that it belongs to these tangential post-synaptic GABAergic neurons. In the MBU, this neuron clearly innervates more than one microglomerulus (Figure 2). This observation, together with the high number of GABAergic neurons connecting to these microglomeruli (Figure 3), indicates that each microglomerulus is invaded by several post-synaptic GABAergic neurons. Very similar GABAergic tangential neurons were previously described in the locust as the source of post-synaptic profiles of the synaptic microglomeruli in the LBU and MBU (Träger et al., 2008).

Apart from the GABAergic varicosities, we also identified some serotonergic varicosities in the LBU and MBU (Figure 4A). Double synapsin/5HT staining suggests that some of these serotonergic varicosities are connected to the large pre-synaptic elements of the microglomeruli (Figure 4B). Thus, these pre-synaptic elements could receive modulatory input from the CBU, CBL and other protocerebral regions via the serotonergic broad field neurons that are the source of these bleb-like terminals (Figures 4C–F). Although a considerable portion of the serotonin-reactive varicosities does not appear to be directly connected to the large pre-synaptic microglomerular profiles (Figures 4A,B), serotonin released by these structures might modulate the post-synaptic microglomerular profiles because these were not stained with the anti-synapsin serum.

### The Possible Roles of Synaptic Microglomeruli in the LBU and MBU

The microglomerular synaptic structures in the LBU and MBU are probably part of the neural circuitry processing sky compass cues in the honeybee brain, as suggested by studies on locusts. Previous studies have shown that the AOTU-LUC of bees receives input from the dorsal rim area (DRA) of the compound eye via the dorsalmost region of the medulla, which is responsible for processing the sun azimuth and the polarized light pattern in the sky (Mota et al., 2011; Pfeiffer and Kinoshita, 2012; Zeller et al., 2015). Thus, the output neurons from the AOTU-LUC that form the large pre-synaptic glomerular profiles in the LBU and MBU, as well as the post-synaptic neurons connecting these glomerular structures to the CBL, might process sky compass information. In the locust, the sky compass pathway connecting the DRA to the medial protocerebrum is similar to the neural pathways described here (Vitzthum et al., 2002; Homberg et al., 2003; Pfeiffer et al., 2005; Träger et al., 2008; Homberg et al., 2011; Pfeiffer and Homberg, 2014). Comparable neural pathways were also recently described in bumblebees (Pfeiffer and Kinoshita, 2012), monarch butterflies (Heinze and Reppert, 2011; Heinze et al., 2013) and desert ants (Schmitt et al., 2016). In the locust, neurons connecting the lower unit of the AOTU to the LBU and MBU, and these two regions to the CBL, are sensitive to polarized light (Vitzthum et al., 2002; Pfeiffer et al., 2005; Pfeiffer and Homberg, 2007; Heinze and Homberg, 2009; Heinze et al., 2009). Similar polarization sensitive neurons involving the LBU and MBU were also described in the cricket (Sakura et al., 2008) and the monarch butterfly (Heinze and Reppert, 2011; Heinze et al., 2013). Although such a polarization sensitivity has not yet been demonstrated in the equivalent neurons of the bee brain, parallels in their morphology and connectivity with respect to those of the locust, cricket and monarch butterfly strongly suggest that they participate in polarized-light processing.

Microglomerular synaptic clusters in the LBU and MBU might also participate in visuospatial detection, learning and memory, as suggested by studies on fruit flies. Mutants of *D. melanogaster* with specific defects or ablations of neuronal subsets in CB structures exhibit varying degrees of learning and memory impairment in visuospatial tasks (Liu et al., 2006; Neuser et al., 2008; Wang et al., 2008; Pan et al., 2009; Ofstad et al., 2011). Here we show that neurons from the microglomerular clusters in the LBU and MBU are highly connected to the honeybee CBL. In fruit flies, the structure analogous to the CBL is the ellipsoid body (Ito et al., 2014; Pfeiffer and Homberg, 2014), which has often been related with visuospatial tasks (Neuser et al., 2008; Ofstad et al., 2011). Microglomerular structures equivalent to the ones described in the present work have been identified in the LX bulbs of *Drosophila* (Jenett et al., 2012). In this insect, ring neurons with dendrites connecting to microglomerular synaptic structures of the bulbs and axon terminals in the ellipsoid body participate in visual feature detection (Seelig and Jayaraman, 2013). These neurons present retinotopically organized receptive fields that are similar to those of simple cells in the vertebrate primary cortex, with strong orientation tuning properties, some degree of direction-selectivity and a high degree of stereotypy (Seelig and Jayaraman, 2013). Physiological responses of dendrites in the microglomerular structures of the fly bulbs were not clearly modulated by walking and flying behaviors, thus suggesting that these neurons do not directly perform motor coordination, but probably provide downstream motor circuits with relevant visual information for motor decisions (Seelig and Jayaraman, 2013). Further studies should analyze whether neurons connecting the LBU and MBU microglomeruli with the CBL in the bee brain are also involved in visual-feature detection and learning.

Despite having similar aspects, the LBU and MBU microglomeruli differed in their glomerular ultrastructure, roundness and pre-synaptic volumes. Furthermore, a recent study showed that LBU microglomeruli receive visual input mainly from the AOTU-LU, whereas the MBU microglomeruli receive input mainly from the AOTU-VLU (Zeller et al., 2015). Therefore, pre-synaptic neurons in the LBU and MBU microglomeruli are connected to different subunits of the AOTU-LUC and present different volumes in their large cup-shaped terminals, suggesting that they have different visual processing functions. The fact that the number of synaptic microglomeruli is higher in the MBU compared to the LBU further supports an integrative role of this structure in visual-information processing compared to the LBU.

### Synaptic Properties and Neural Plasticity in LBU and MBU Microglomeruli

In the bee, each LBU and MBU microglomerulus is composed of a large pre-synaptic profile that embraces numerous smaller post-synaptic profiles. This synaptic organization has previously been described in locusts (Träger et al., 2008), and resembles to some extent the giant synapses described in the mammalian auditory system, the so-called calyx and endbulb of Held (Ryugo et al., 1996; Sätzler et al., 2002). In these giant synapses (up to 30 μm diameter), the large cup-shaped pre-synaptic profile that encloses the post-synaptic structure provides a diffusion barrier and releases numerous vesicles of neurotransmitter following an action potential, thus accounting for a high spike time precision (Schneggenburger and Forsythe, 2006; Borst and Soria van Hoeve, 2012). The synaptic structure of microglomerular clusters in the LBU and MBU of insects may also constitute an adaptation for strong and fast synaptic transmission (Träger et al., 2008). Given that the pre-synaptic volumes were larger in the MBU than in the LBU microglomeruli, the function of MBU microglomeruli could demand a higher spike-time precision when compared to that of LBU microglomeruli.

Smaller microglomerular synaptic structures (2–3 μm in honeybees; Ganeshina and Menzel, 2001) have been studied in the calyces of the MBs (Groh and Rössler, 2011). These calycal microglomeruli are composed of a large central pre-synaptic bouton from a projection neuron (PN) surrounded by post-synaptic profiles from Kenyon cell dendrites (Ganeshina and Menzel, 2001; Yasuyama et al., 2002; Groh and Rössler, 2011). Although the arrangements of the pre- and post-synaptic elements in these calycal microglomeruli are inversed (post-synaptic elements embrace the pre-synaptic one) in relation to the microglomeruli described for the LX of honeybees (the pre-synaptic element embraces post-synaptic ones), both types share a single pre-synaptic profile that makes synaptic contacts with numerous post-synaptic profiles. MB microglomeruli exhibit a remarkable structural plasticity depending on postembryonic brood care, age, sensory experience (olfactory and visual) and memory formation (reviewed in Rössler and Groh, 2012). Visual-related structural plasticity has been described in MB microglomeruli of honeybees and ants (Krofczik et al., 2008; Stieb et al., 2010, 2012; Yilmaz et al., 2016). Given that the microglomerular synaptic clusters in the MBU and LBU are probably involved in sky-cue and/or visuospatial processing, it is worth determining if visual experience also promotes structural and functional plasticity in these structures. A recent work demonstrated that foragers have significantly more synaptic LX microglomeruli than interior workers of the desert ant *Cataglyphis fortis* (Schmitt et al., 2016). These authors found that the increase of LX microglomeruli in foragers was not age-related, but depends on light exposure, with a more pronounced effect triggered by UV-spectrum exposure. The degree of variability that we found in the number of MBU and LBU microglomeruli of forager honeybees, as well as in the volume of their pre- and post-synaptic profiles could reflect some degree of plasticity due to factors like the ones mentioned above. Further studies should analyze whether MBU and LBU microglomeruli of the honeybee brain are subject to functional and structural plasticity and characterize its determinants.

### Experimental Perspectives on Microglomerular Physiological Studies

The anatomical description of MBU and LBU microglomeruli provided in the present study opens new research avenues to understand the physiology and function of these structures in the honeybee. Electrophysiological recordings of pre-synaptic and post-synaptic neurons building these microglomeruli are necessary to determine the functional properties of each neuron category, as achieved for equivalent neurons of the desert locust, the cricket and the monarch butterfly brain (Vitzthum et al., 2002; Pfeiffer et al., 2005; Pfeiffer and Homberg, 2007; Sakura et al., 2008; Heinze and Homberg, 2009; Heinze et al., 2009, 2013; Heinze and Reppert, 2011). The particularly large cup-shaped morphology of LBU and MBU pre-synaptic profiles makes it attractive to investigate pre-synaptic mechanisms of neurotransmission in the insect central nervous system.

At the circuit level, calcium-imaging of neurons connecting to the microglomeruli could also allow understanding if and how visual attributes such as features, shapes, azimuth or light polarization are spatiotemporally coded in the LBU and MBU of honeybees, as recently performed in fruit flies (Seelig and Jayaraman, 2013). Although the possibility of using mutants with genetically targeted neural populations expressing a calcium-indicator is still not available in honeybees, our team has recently developed a technique that allows recording *in vivo* calcium signals from visual neural circuits of the honeybee brain (Mota et al., 2011, 2013). The combination of pharmacology with electrophysiology or calcium-imaging at the level of the LBU and MBU synaptic microglomeruli may yield new insights into the different forms of neurotransmission modulating microglomerular function. Taken together, these approaches offer exciting perspectives for achieving an integrative comprehension of central visual processing and learning in the honeybee brain.

## Author Contributions

TM, SK, GG, and MG designed research; TM, SK, ACD and DL performed research; TM, SK and ACD analyzed data; TM, SK, GG and MG wrote the article.

## Funding

We thank the generous support of the Human Frontiers Science Program (HFSP) and of the Institut Universitaire de France (IUF) to MG. This work was also funded by the Minas Gerais State Research Foundation (FAPEMIG: APQ 00299-13 to TM), the Brazilian Council for Scientific and Technological Development (CNPq: 457718/2014-5 to TM), the French Research Council (CNRS) and the University Paul Sabatier of Toulouse.

## Conflict of Interest Statement

The authors declare that the research was conducted in the absence of any commercial or financial relationships that could be construed as a potential conflict of interest.
